# Adipokines as potential prognostic biomarkers in patients with acute knee injury

**DOI:** 10.3109/1354750X.2014.948914

**Published:** 2015-05-26

**Authors:** Stefan Kluzek, Nigel K. Arden, Julia Newton

**Affiliations:** ^a^ Nuffield Department of Orthopaedics, Rheumatology and Musculoskeletal Sciences, University of Oxford, Oxford, UK; ^b^ Oxford NIHR Musculoskeletal Biomedical Research Unit, Nuffield Department of Orthopaedics, Rheumatology and Musculoskeletal Sciences, University of Oxford, Oxford, UK; ^c^ MRC Lifecourse Epidemiology Unit, University of Southampton, Southampton General Hospital, Southampton, UK

**Keywords:** Bone repair, cytokines, growth factors, inflammatory mediators, obesity/diabetes, osteoarthritis

## Abstract

This review considers adipokines as predictive biomarkers for early onset post-traumatic knee osteoarthritis (KOA). Serum concentrations of leptin and resistin can predict radiographic changes and are elevated in early KOA, with higher leptin concentrations independently associated with more severe knee changes. Plasma concentrations of resistin are chronically elevated after injury. Leptin, resistin, chemerin and vistfatin induce catabolic enzymes associated with cartilage degeneration. Available literature on adipokines in post-traumatic KOA pathogenesis suggests that they could contribute to risk prediction of early onset post-traumatic KOA. Further research is needed to further understand the association between adipokines, synovitis and long-term outcomes in this population.

## Context

Adipokines are cytokines produced by white fat tissue in response to a positive energy balance determined by food intake. Obesity is a model of chronically elevated circulating levels of pro-inflammatory adipokines. The biomechanical aspects of increased body mass index (BMI) cannot explain the high incidence of osteoarthritis (OA) in the non-weight-bearing joints. Current concepts suggest that adipose tissue-derived cytokines can increase inflammatory responses to mechanical insults and result in fast progression to joint failure (Oliveria et al., 1999). This article will review current literature looking at the role of adipose tissue-associated inflammation in the pathogenesis of post-traumatic knee osteoarthritis (KOA) in the context of its impact on interactions between cartilage, synovium and subchondral bone.

## Methods

A comprehensive search of electronic databases was carried out on Pubmed, the Cochrane Central Register of Controlled Trials, CINAHL and reference lists of relevant articles. The PubMed literature database was searched using MeSH index terminology. The following broad search terms were included: knee, post-traumatic and/or OA in combination with obesity, adipokine or adiponectin. This was complimented by a hand search of OA journals. This search was limited to human and animal studies in the English language. Limits to publication date applied between January 1985 and May 2014.

### Systemic and local inflammation in post-traumatic knee osteoarthritis

Systemic inflammation measured by IL-6 has been shown to be an independent predictor of radiographic KOA in healthy, middle-aged British women (Livshits et al., 2009). Knee injury and obesity are strongly associated with the development of KOA (Anderson & Felson, 1988; Davis et al., 1989; Felson et al., 1988; Jiang et al., 2012; Lohmander et al., 2009; Roos, 2005). Blagojevic et al. have estimated, through a meta-analysis, that the overall pooled odd ratio (OR) for overweight and obese individuals is 2.63 (95% CI 2.28–3.05) and 3.86 (95% CI 2.61–5.70) (Blagojevic et al., 2010) for those with previous knee trauma. Obesity is characterized by high white adipose tissue (WAT) mass and associated with a state of chronic low-level inflammation. Anterior cruciate ligament (ACL) or meniscal injuries are among the strongest risk factors for early development of post-traumatic KOA (Englund et al., 2003; Neuman et al., 2008; Roos, 2005; von Porat, 2004). They represent a great model for the development of KOA, with well-defined early onset of disease. The incidence of all knee injuries is greatest in males under 30 years of age (Gianotti et al., 2009; Majewski et al., 2006). Joint injury may lead to a mild-to-moderate local inflammatory reaction (Pessler et al., 2008) and inflamed knee synovium has been shown to be associated with more severe cartilage degeneration over the course of a year (Ayral et al., 2005). High systemic markers of inflammation have been previously reported in patients with knee trauma (Mendias et al., 2013).

Historically, the development of post-traumatic KOA in humans with ACL-deficient knees has been mainly attributed to recurrent episodes of knee instability. Primary ACL reconstruction, aimed at restoring mechanical stability, has not so far been proven to decrease the incidence of KOA or improve long-term function in cohort or randomised controlled studies (Barenius et al., 2014; Frobell et al., 2010, 2013; Lohmander et al., 2004; von Porat, 2004). It implies that another mechanism might be responsible for driving joint degeneration after initial trauma associated with instability.

Elevated BMI has been one of the strongest predictors of early KOA development after ACL and/or meniscal injuries, with an adjusted OR between 1.17 and 3.1 (Barenius et al., 2014; Kessler et al., 2008; Lebel et al., 2008; Li et al., 2011). Along with knee injury, other risk factors for the development of KOA, age and female gender, are also associated with increased systemic inflammation and a relatively higher percentage of fat tissue (Pirkola et al., 2010).

The proposed overlapping common mechanism is an adipose tissue-promoted inflammation. There is now increasing evidence that, at least in a subset of the patients, both inflammatory reactions of synovium and high adipokine concentrations may be prevalent causes of early deterioration of the knee joint (Guermazi et al., 2014; Karvonen-Gutierrez et al., 2014; Roemer et al., 2011; Sokolove & Lepus, 2013). In animal models, the effect of even extreme obesity on development of KOA is diminished in animals without a functional main adipokine pathway (Griffin et al., 2009).

Adipose tissue produces a series of chemicals that have been linked with the regulation of systemic and organ-specific inflammation, organ damage and chronic diseases like diabetes (Bozaoglu et al., 2007; Fantuzzi, 2005; MacDougald & Burant, 2007). It has been postulated that increased systemic inflammation and activity of organ-specific adipose stores promotes local inflammation. It has also been suggested that, on the joint level, this results in prolonged synovitis and affects the cartilage’s ability to maintain an equilibrium between synthesis and degeneration of cartilage constituents (Bao et al., 2010; de Boer et al., 2012; Perruccio et al., 2014).

### Signals from adipose tissue

Lipogenesis and lipolysis are two primary metabolic activities of WAT (Cristancho & Lazar, 2011). Both processes are regulated by endocrine and neural mechanisms. The discovery of the first adipose tissue hormone, leptin, and an increased interest in the endocrine functions of WAT have allowed for the identification of cytokines known collectively as adipokines (Ahima, 2006). The majority of these have been linked with lipid metabolism but also with inflammation. Adipokines associated with inflammation include classical pro-inflammatory agents (IL-6, TNF-α) and tissue specific agents such as leptin, adiponectin, Vaspin, Resistin, chemerin, visfatin and adipsin. IL-6 produced by adipose tissue contributes to around a third of circulating IL-6 and is strongly associated with increasing obesity (Proenca et al., 2014).

Osteophytes are a structural hallmark of KOA and are associated with knee pain. Individuals with higher BMIs have an increased risk of fast cartilage loss (Roemer et al., 2009) and report more severe knee pain (Weiss, 2014). High ratio of fat mass to skeletal muscle mass is also positively associated with more severe KOA structural changes on magnetic resonance imaging (MRI) (Visser et al., 2014). Some adipokines have been linked with development of more severe knee changes (Karvonen-Gutierrez et al., 2014) and more painful KOA (Perruccio et al., 2014). Similarly, patients with a higher BMI are more likely to develop radiographic KOA after anterior crucial ligament reconstruction (Li et al., 2011). Secreted adipokines have also been shown to affect bone cell differentiation and can potentially impact on bone remodelling and bone formation (Bartell et al., 2011; Hamrick et al., 2004; Muruganandan et al., 2010, 2013). Moreover, bone marrow mesenchymal stem cells (MSCs) can differentiate, not only into bone forming osteoblasts, but also into adipocytes that can competitively suppress intracellular osteogenic signals (Muruganandan & Sinal, 2014).

### Leptin

Leptin is a hormone that is produced in proportion to WAT mass and has been widely discussed in literature in the context of pro-inflammatory properties in OA. Its concentration is greater in women but it has not been entirely explained by either adipose load or sex hormone concentrations (Licinio et al., 1998). Apart from decreasing appetite, leptin promotes neutrophil mobilisation, cytotoxic lymphocyte and macrophage activation (Carbone et al., 2012). It also enhances production of MMP-1, MMP-3 and MMP-13 in human KOA cartilage (Koskinen et al., 2011), resulting in a progressive articular cartilage degeneration. The effect of leptin shows sex dimorphism in the association of its effect with KOA. Higher levels have been shown to be an independent predictor of MRI and radiographic knee changes associated with OA, but mainly in women.

#### Animal studies

Leptin has been shown to have both a strong anabolic and catabolic function in chondrocytes. The anabolic function occurs through the induction of IGF-1 and TGFβ1 synthesis (Dumond et al., 2003), while the catabolic function occurs by inducing MMP1 and MMP13 expression with a concomitant activation of STAT, MAPK, Akt and NF-κB signalling pathways (Hui et al., 2012). Diet-induced obesity in mice increases serum concentrations of leptin and KOA scores (Griffin et al., 2012). Moreover, extreme obesity in animals with impaired leptin signalling (leptin or leptin receptor deficient) does not cause increased incidence of KOA (Griffin et al., 2009). Leptin also affects bone metabolism and central injection (intra-cerebroventricular administration) leads to enhanced bone formation in a mutant leptin-deficient mouse model (ob/ob mice) (Bartell et al., 2011).

#### Human studies

Leptin receptors have been found to be expressed in articular chondrocytes (Figenschau et al., 2001) and modulate expression of canonical Wnt signalling receptors (Ohba et al., 2010). Synovial fluid (SF) and serum concentrations correlate with BMI in patients with established KOA, while SF/serum ratio is higher in early KOA (de Boer et al., 2012; Dumond et al., 2003; Staikos et al., 2013). Unlike other adipokines, leptin concentrations in SF are similar or higher than serum concentrations (Presle et al., 2006). Higher plasma concentrations have been associated with greater painful joint burden (Perruccio et al., 2014), with prevalent and incident KOA (OR 1.31, 95% CI 1.21–1.41), over a 10-year period in middle-aged women (Karvonen-Gutierrez et al., 2013). In a 5-year cohort study with very early-stage KOA, leptin has been shown to be associated with higher levels of systemic markers of synovial and cartilage metabolism and progression but not with the incidence of radiographic KOA (Van Spil et al., 2012). It has been cross-sectionally and longitudinally associated with reduced cartilage thickness (Stannus et al., 2013) and, interestingly, both obesity and female gender effects have mainly been related to leptin (Ding et al., 2008). Baseline levels have been associated with increased levels of bone formation biomarkers (over 2 years) in patients with established KOA (Berry et al., 2011). These have been correlated with larger osteophyte formation, synovitis and effusion on MRI (Karvonen-Gutierrez et al., 2014). Recently, in women, higher leptin has been associated with ∼30% higher risk of having structural KOA (Karvonen-Gutierrez et al., 2012).

In summary, serum leptin levels have been positively linked with KOA on a molecular level and *in vivo*, both in animal models and human clinical studies. This review has not identified any studies looking at the role of leptin specifically in the development of post-traumatic KOA.

### Adiponectin

This adipokine modulates insulin sensitivity and inflammation. It has been shown to have both anti- and pro-inflammatory properties, but its pivotal role is associated with an inhibitory effect of proinflammatory cytokines, such as tumour necrosis factor and interleukin-6 (IL-6). It is also associated with the induction of expression of anti-inflammatory proteins, including IL-10 and IL-1 receptor antagonist (Kumada et al., 2004; Tsatsanis et al., 2005; Wolf et al., 2004). Females have higher circulating levels of adiponectin than males (Combs et al., 2003). Visceral adipocytes are mainly responsible for adiponectin’s systemic levels and, paradoxically, those are reduced in obesity (Arita et al., 2012). The transcription of adiponectin in adipocytes is suppressed by TNF and IL-6, which might explain the lower levels of serum adiponectin in obese individuals (Bruun et al., 2003; Fasshauer et al., 2003; Maeda et al., 2002). Two types of adiponectin receptor have been identified: AdipoR1, which mainly activates the AMPK phosphorylation pathway and AdipoR2, which is involved in the activation of PPAR-α (Lee et al., 2008).

#### Animal studies

Adiponectin is effective in reducing the activation of inflammatory pathways, including the NF-κB pathway (Lira et al., 2012). Diet-induced obesity has been shown to significantly increase the severity of post-traumatic KOA in a common inbred strain of laboratory mice. More importantly, levels of synovial inflammation in controlled uninjured knees inversely correlates with systemic levels of adiponectin (Louer et al., 2012).

#### Human studies

Adiponectin has not been detected in healthy cartilage and is up-regulated in patients with OA (Francin et al., 2014). It has been shown to be weakly associated with synovial inflammation in patients with end-stage KOA (de Boer et al., 2012). In human chondrocytes cultures, adiponectin induces MMP-3 (Tong et al., 2011). In patients with hand OA, high levels of adiponectin have been associated with a 70% reduction of joint space narrowing over a 6-year period (Yusuf et al., 2011). Development of early symptomatic KOA has been negatively associated with levels of adiponectin, and positively with high-sensitivity CRP (hs-CRP). Levels of adiponectin have not been shown to predict progression or incidence of radiographic KOA (Van Spil et al., 2012). Decreased levels in both plasma and SF have been shown to be lower in more advanced KOA, indicating that it may have a protective role (Honsawek & Chayanupatkul, 2010). Adiponectin has not been linked with an increase of bone formation biomarkers so far (Berry et al., 2011).

In summary, higher systemic concentrations of adiponectin probably protect against development of KOA in animal models, while some clinical human studies also confirm these properties. Despite this, adiponectin’s role in the regulation of joint inflammation and cartilage homeostasis is still not clear.

### Resistin

The adipocytokine resistin was initially investigated mainly as an insulin resistance inducing factor in mice (Steppan et al., 2001). The receptor for resistin is unknown, but it has strong pro-inflammatory properties and has been shown to trigger the release of other proinflammatory cytokines such as TNF- α, IL-1β and IL-6 (Bokarewa et al., 2005).

#### Human studies

Serum resistin is elevated in obese individuals (Degawa-Yamauchi, 2003) but this correlation is obscured in patients with advanced KOA (de Boer et al., 2012). In humans, resistin correlates better with subclinical inflammation than with insulin resistance (Steppan & Lazar, 2002). Resistin can be detected locally in the synovium of patients with KOA and correlates with histologically defined synovitis (de Boer et al., 2012). Both systemic and SF concentrations are elevated in post-traumatic knees and associated with the release of cytokines and cartilage degeneration (Lee et al., 2009). In a very early symptomatic KOA cohort, resistin levels have been shown to be positively associated with synovial biomarkers (sPIIINP) and high-sensitivity CRP but, more interestingly, with the incidence of radiographic KOA (Van Spil et al., 2012). Such findings are independent of BMI and testing hs-CRP as a potential confounder in this association has suggested that those processes are independent.

#### Adipsin

Adipsin is involved in triglyceride metabolism but its effect on cartilage and the rest of the knee joint is not yet known, including any role in the modulation of synovitis after joint trauma. Higher adipsin levels have been found in obese individuals (Abu-Farha et al., 2014; Bienertova-Vasku et al., 2014). Lower serum concentrations of adipsin have been associated with greater painful joint burden in patients with end-stage hip and knee OA (Perruccio et al., 2014).

### Chemerin

Chemerin is a novel adipokine that affects adipocyte differentiation and metabolism *in vitro* (MacDougald & Burant, 2007). It acts through G protein-coupled receptor: the chemokine like receptor-1 (CMKLR1, also known as ChemR23) and is expressed by circulating plasmacytoid dendritic cells, tissue-resident macrophages and adipocytes. It has been postulated that chemerin increases macrophage infiltration and activates a local inflammatory response (Goralski et al., 2007). Stimulating human chondrocytes with chemerin results in an increase in phosphorylation of Akt and a probable subsequent activation of MEK1/2. It also further activates the MAPK pathway and increases concentration of TNF-a, IL-1b, IL-6 and IL-8 (Berg et al., 2010). Along with this, it increases Toll-like receptor 4 mRNA and the synthesis of CCL2 in OA synoviocytes (Eisinger et al., 2012). The activated Toll-like receptor 4 leads to the development of an inflammatory reaction involving macrophages. CCL2 has been linked with mediating movement-related pain signalling in the animal model of post-traumatic KOA (Miller et al., 2012).

#### Animal studies

Mice bone marrow–derived osteoblast precursor cells differentiate to adipocytes when stimulated by chemerin (Muruganandan & Sinal, 2014). The increased bone mineralization with chemerin, in the CMKLR1 knockdown model, suggests that this signalling pathway acts, not only to promote adipogenesis, but also to actively suppress osteoblastogenesis.

#### Human studies

Chemerin has been associated with visceral obesity (Shin et al., 2012). High levels have been found to be associated with the activity of inflammatory arthritis (rheumatoid and psoriatic) (Ha et al., 2014; Xue et al., 2012). Chemerin is detected in SF and its higher concentrations are associated with KOA severity (Huang et al., 2012). SF levels of chemerin are similar in patients with KOA, rheumatoid and psoriatic arthritis (Valcamonica et al., 2014). Human chondrocyte cultures from patients with recent ACL injuries and end-stage KOA cells have been positive for both ChemR23 and chemerin staining (Berg et al., 2010). Stimulation of those cultures with chemerin has resulted in a significant elevation of MMP-1, MMP-2, MMP-3, MMP-8 and MMP-13 levels, catabolic enzymes associated with cartilage degeneration.

### Visfatin

Visfatin is called pre-B-cell colony-enhancing factor and is mainly produced by adipocytes in visceral fat and associated with increases in insulin resistance (Fukuhara et al., 2005). It regulates intracellular activity of the NAD-consuming enzymes affecting the production of inflammatory cytokines (TNFα, IL-6 and IL-1β) (Moschen et al., 2010). So far, no specific receptor has been identified, but it is thought that visfatin exerts proinflammatory action by regulating the insulin receptor pathway activity (Jacques et al., 2012). Human chondrocytes stimulated with visfatin induce synthesis of MMP-3 and MMP-13 (Gosset et al., 2008).

#### Human studies

Higher levels are observed in subjects with broader waists (Bienertova-Vasku et al., 2014) and associated with higher triglicerides but lower HDL (Abu-Farha et al., 2014). Patients with rheumatoid arthritis have higher serum levels of visfatin than healthy controls (Otero et al., 2006). Vistafin has been also shown to be produced and stored by synovial membrane, cartilage and the subchondral bone in patients with KOA (Laiguillon et al., 2014).

## Summary

Animal research in this field, assessing the role of pro-inflammatory cytokines related to adipose tissue in the development of post-traumatic OA (PTOA), is very promising. Animals with impaired leptin pathways do not develop KOA, even in the presence of extreme obesity, indicating that increased loading cannot explain cartilage degeneration alone. Higher leptin levels have been associated with more severe progression of knee joint degeneration.

Leptin, resistin, chemerin and vistfatin have all been shown to induce chondrocyte production of matrix metalloproteinases. Based on the available literature on the involvement of adipokines in post-traumatic KOA pathogenesis, it is plausible that adipokines (especially leptin in women), resistin and chemerin (in men and women) may, in the future, contribute to a risk model of early development of KOA after ACL and/or meniscal injury. The relative activity of the various adipokines with respect to their effects on the synovium, bone and cartilage after knee trauma remains to be determined. Local changes in adipokine concentrations may have important pathophysiological implications for cartilage homeostasis but, so far, only leptin seems to correlate well with SF concentration and the systematic elevation of resistin levels has been proven to reflect SF concentration after joint injury.

This review identifies the need for prospective cohort studies to investigate the role of serum and SF adipokines as potential biomarkers for early post-traumatic OA in patients with acute ACL and/or meniscal injuries. Long-term follow-up will help to identify those at risk of a poor outcome and identify possible therapeutic targets in this pathway.
